# Investigating Blood Biomarkers That Can Facilitate the Diagnosis of Meningitis—A Systematic Literature Review

**DOI:** 10.3390/ijms26041427

**Published:** 2025-02-08

**Authors:** Jakub Marek Baran, Adrianna Porębska, Magdalena Lesisz, Katarzyna Polak, Olga Grodzka, Izabela Domitrz

**Affiliations:** 1 Department of Neurology, Faculty of Medicine and Dentistry, Medical University of Warsaw, 01-809 Warszawa, Poland; s079907@student.wum.edu.pl (J.M.B.); s086195@student.wum.edu.pl (A.P.); s084636@student.wum.edu.pl (K.P.);; 2 Doctoral School, Medical University of Warsaw, 02-091 Warsaw, Poland

**Keywords:** biomarker, infectious diseases, inflammation, lumbar puncture, meninges, meningitis, sepsis

## Abstract

Meningitis is an inflammation of the meninges that can sometimes be a life-threatening disease. Therefore, fast and proper diagnosis with the implementation of adequate treatment is crucial in its management. Treatment depends on etiology, which can be viral, bacterial, fungal, and parasitic. Diagnosis is based on thorough clinical examination with a performance of lumbar puncture in the case of meningitis suspicion. This procedure, however, remains invasive with several contraindications and a need for a patient’s consent, which is not always given due to the patient’s fear of it, for instance. Thus, this systematic review aimed to summarize the available literature on the topic of blood biomarkers in meningitis differentiation. A selection process was performed by two authors independently in accordance with the Preferred Research Items for Systematic Reviews and Meta-analyses. Two databases were screened. It led to the identification of 863 articles, of which 43 were eventually included in the systematic review. The analysis resulted in identifying blood biomarkers in both adult and pediatric meningitis. Most studies focused on inflammatory markers, such as C-reactive protein and procalcitonin, from which procalcitonin showed better utility. Among other analyzed molecules were, for instance, interleukins, apolipoproteins, and microRNAs. Moreover, many researchers suggested that combining biomarkers or implementing novel technologies may lead to the best accuracy. However, many suggested methods lack validation, which stands in the way of making them widely used.

## 1. Introduction

Meningitis is an inflammation of the meninges, including the dura mater, arachnoid mater, and pia mater, which cover the brain and the spinal cord [1]. Due to the anatomical correlation of the meninges with the brain cortex and the parenchyma, those structures may also be affected by this process [2]. This state can be caused by several viral, bacterial, fungal, and parasitic factors, although noninfectious diseases also contribute to the occurrence of meningitis [2,3]. The most common form of this disease is aseptic meningitis, with viruses as the leading cause. What is worth mentioning is that in this review, some studies compare bacterial meningitis (BM) to aseptic meningitis, while others to viral meningitis (VM); notably, those terms, although sometimes similar, are indeed not the same [4]. Among the viral factors, Enteroviruses are responsible for approximately 85–95% of the cases [5,6]. Despite the high prevalence, most VM is usually benign and self-limiting. Compared to them, BM is considered the most severe form of meningitis with high mortality [1]. According to the World Health Organization (WHO), despite the existence of vaccines against Streptococcus pneumoniae, Neisseria meningitidis, and Hemophilus influenzae, those three pathogens, along with Streptococcus agalactiae, remain responsible for more than half of the deaths from meningitis globally [7].

The diagnosis of meningitis requires a thorough history of the patient and a physical examination, with the application of specific clinical tests to indicate meningeal irritation such as neck stiffness, tripod phenomenon, Kernig’s sign, or Brudzinski’s I and II signs [8]. Those might be insensitive but sometimes highly specific, and their presence should always raise suspicion [5,8]. If the physician suspects meningitis, a lumbar puncture should be performed to obtain the material for cerebrospinal fluid (CSF) analysis [9]. This procedure remains the most appropriate method of verifying the diagnosis and determining the etiology of meningitis [9]. Noteworthily, in the case of elevated intracranial pressure, performing the lumbar puncture may lead to cerebral herniation, which limits the application of this procedure [6]. Sometimes, a clinician has to balance costs and benefits, analyzing indications and contraindications to the lumbar puncture. In such cases, additional tests indicating increased intracranial pressure, such as brain neuroimaging or fundoscopy, may help in assessing the risk of herniation. Aseptic meningitis usually follows a benign clinical course and is self-limiting; however, in some cases, it might require hospitalization and specific therapy [5,10]. Such an example is meningitis resulting from Herpes simplex viruses (HSVs) and the application of acyclovir is recommended to avoid the serious neurologic sequela [5,6]. Noteworthily, the most common neuroinfection caused by HSV is encephalitis, which, alone or together with meningeal infection as meningoencephalitis, has a much poorer prognosis than HSV meningitis [11,12]. If BM is suspected, the treatment with broad-spectrum antibiotics should be applied immediately after the lumbar puncture, followed by the adjustment of antibiotics after obtaining the CSF culture results [13]. Such demeanor may prevent the deterioration of the clinical state of the patient and diminish the risk of mortality [6]. Therefore, the systematic review aims to summarize the current knowledge on the topic of blood biomarkers to facilitate meningitis differential diagnosis.

## 2. Methods

The systematic review was written following the Preferred Reporting Items for Systematic Reviews and Meta-Analyses (PRISMA 2020) guidelines [14]. The study protocol was registered in PROSPERO, the International Prospective Register of Systematic Reviews (ID: CRD42024607277).

### 2.1. Inclusion and Exclusion Criteria

To indicate only appropriate studies, inclusion and exclusion criteria were applied. The allowed study designs were randomized controlled trials and observational studies (including cohort, case–control, and cross-sectional studies). To be included in the review, studies had to cover the issue of blood biomarkers in meningitis, either in adults or children. Furthermore, only articles written in English were allowed.

In comparison, study designs such as reviews, meta-analyses, case reports, case series, editorials, and commentaries were not considered. Additionally, conference abstracts were not allowed. Articles not covering the review topic or written in languages other than English were excluded.

### 2.2. Selection Process

**Figure 1:**
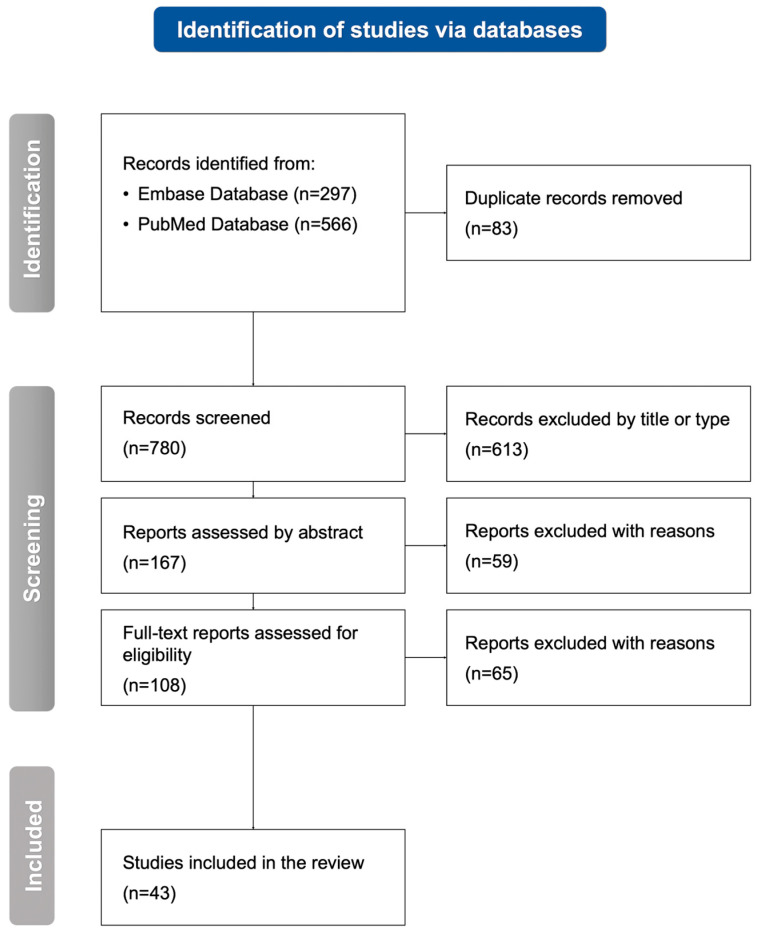
A flowchart of the selection process performed following the Preferred Reporting Items for Systematic Reviews and Meta-Analyses (PRISMA 2020) guidelines. *n*, number of studies.

The selection (Figure 1) was performed independently by two authors (J.B. and A.P.). Each discrepancy was additionally reviewed and re-assessed by the third author (O.G.) to avoid bias. All the authors assessed the studies carefully to find and exclude research with the highest risk of bias if necessary. Moreover, the quality of the articles has been evaluated using the methodological quality appraisal tool [15], which included the criteria of significant aspects of research methodology: outcome measures, background or literature review, sample description, study design and methodology, and conclusions. Each criterion was evaluated for 0 or 1 point. Studies assessed as low-quality with a score of 2 or fewer points were excluded (Supplementary Materials Table S1).

Two databases were screened with a search strategy as follows: (meningitis) AND (bacterial OR viral OR fungal) AND (biomarker) AND (diagnosis) AND (blood OR serum OR plasma). The initial search identified 863 records (297 from the PubMed Database and 566 from the Embase Database). After duplicate removal, 780 studies were assessed, and 613 were excluded because of inappropriate titles or types. Further, based on an abstract analysis of 167 reports, 59 did not meet the inclusion criteria. A final search of 108 full-text articles led to the exclusion of 65 reports. Eventually, 43 appropriate studies were included in the systematic review.

Data extraction was conducted systematically using predefined criteria to ensure accuracy and consistency, capturing key details such as study design, population characteristics, interventions, outcomes, and main findings. Data synthesis followed a structured approach, incorporating narrative synthesis and thematic analysis to integrate and interpret the findings across studies in a comprehensive and meaningful manner.

## 3. Results and Discussion

Considering the differences in the symptoms, course, treatment, and outcomes of meningitis in various age groups, the review focuses separately on studies conducted on adult populations and those in pediatric populations. The age was precisely defined in the tables if given. When the studied population was over 18 years old without more specific information, patients were described as adults. Finally, cases in which the study populations were not narrowed to a specific age group are described in Section 3.6. in the current arrangement. The variables that the work has focused on were the levels or expression of blood biomarkers in differentiation between meningitis etiologies with additional information such as accuracy, sensitivity, and specificity. The majority of research focused on the two most common indicators of inflammation: procalcitonin (PCT) and C-reactive protein (CRP); however, other biomarkers have also been considered and adequately outlined. Considering this observation, CRP and PCT were discussed in distinct paragraphs from others.

### 3.1. Procalcitonin and C-Reactive Protein as Blood Biomarkers in Adult Meningitis

Several research studies have focused on a correlation between elevated levels of blood PCT and BM in adults [16,17,18,19,20], showing PCT may be considered a possible blood biomarker in BM diagnosis (Table 1). The study by Shen et al. presented a positive correlation between blood PCT levels and PCT levels, leukocytes, and proteins in CSF [16]. The levels of PCT in both serum and CSF were significantly elevated in patients with BM. Interestingly, the area under the receiver operating characteristic (ROC) curve for serum PCT in diagnosing BM was 0.96 (95% confidence interval [CI]: 0.93–1.00), which is considerably higher than the PCT level in CSF (0.90, 95% CI: 0.83–0.96). This indicates a better diagnostic value of serum PCT compared to CSF PCT among patients suspected of having BM. These findings are corroborated by the study by Viallon et al., which demonstrated that the most differentiating parameters in diagnosing BM are serum PCT and CSF lactate levels [17]. Moreover, an advantage of sensitivity (95% vs. 94%), specificity (100% vs. 92%), negative predictive value (100% vs. 99%), and positive predictive value (97% vs. 82%) was observed for serum PCT testing. Zhang et al. analyzed three groups of meningitis patients: suppurative, VM, and tuberculous (TBM), and compared them to each other, as well as the control group with migraine or epilepsy but without any infection [18]. The group of suppurative meningitis presented significantly higher levels of serum PCT than the three other groups. Karan et al., on the other hand, compared the sensitivity and specificity in diagnosing BM of PCT either in CSF or in plasma and showed no statistically significant differences between those body fluids [19]. Serum PCT reached a sensitivity of 83.3% and specificity of 86.5%, while CSF PCT presented a sensitivity and specificity of 86.7% and 92.3%, respectively. Finally, the study by Alnomasy et al. showed the highest diagnostic accuracy of protein levels in CSF for differentiating between bacterial and VM [20]. However, the authors emphasized that the combination of CSF protein level testing and serum PCT significantly improved predictive accuracy for anticipating BM.

C-reactive protein was the second inflammatory indicator found to have a potential diagnostic utility in differentiating between aseptic and BM. The study by Takada et al. evaluated CRP levels in patients without meningitis, those with BM, and those with aseptic meningitis [21]. The median CRP level was 5.6 mg/dL for patients without meningitis, 0.2 mg/dL in patients with aseptic meningitis, and 21.7 mg/dL in patients with BM. Interestingly, patients without meningitis showed higher CRP levels than those with aseptic meningitis. In the study by Morales-Casado et al., the usefulness of PCT and CRP testing in differentiating BM from VM (VM) was assessed [22]. The levels of PCT and CRP differed significantly between the bacterial and viral etiologies. However, serum PCT was also shown to be more effective in detecting BM than CRP [5].

### 3.2. Other Blood Indicators of Adult Meningitis

Apart from PCT and CRP, which are well-known inflammation indicators, other putative molecules have been constantly suggested [23,24,25,26,27,28,29]. Serum glial protein S100 calcium-binding protein B (S100B) has been extensively investigated to determine whether it could serve as an effective biomarker for identifying patients at risk of severe brain damage [23]. Grønhøj et al. conducted a study comparing S100B levels in adult patients with severe community-acquired acute BM to healthy, non-meningitis controls [21]. The investigation revealed a significant increase in S100B levels in the former group compared to the latter. Interestingly, the highest values were observed at the beginning of hospitalization. This demonstrates the utility of S100B in diagnosing BM rather than predicting the course or character of this illness. In addition, Lins et al. conducted an independent study investigating S100B levels in CSF and serum from patients with BM, VM, and neuroborreliosis, in contrast to individuals without CNS infection [24]. Similarly to the study mentioned before, the S100B levels were significantly elevated in patients with cerebral damage, with the highest levels observed at the beginning of hospitalization. It was also found that S100B concentrations in both CSF and serum were significantly higher in BM compared to VM, potentially reflecting the greater severity of blood–CSF/brain barrier disruption in BM. Moreover, the hypothesis that S100B could be a potential serum biomarker for meningitis diagnosis was investigated in another study. Undén et al. compared S100B levels among patients with various CNS infections, including bacterial and VM [25]. It was demonstrated that 73% of the patients with BM had elevated S100B levels compared to only 7% of the patients with VM. However, despite the higher S100B levels in BM compared to VM, the highest mean levels of this biomarker were observed in viral encephalitis, nearly twice as high as in BM (0.58 mg/L). Moreover, elevated S100B levels were found in 25% of the cases of extracerebral infections. Although these values appeared less frequently compared to meningeal infections, the variability and inaccuracy of S100B may limit its exclusive use for meningitis diagnosis.

Neurogranin is a postsynaptic protein involved in neurotransmission that can cross a damaged blood–brain barrier. Consequently, it has been investigated as a potential biomarker for acute brain injury. Canturk et al. conducted a case–control study to evaluate the efficacy of neurogranin in diagnosing central nervous system (CNS) infections, comparing patients with confirmed BM to healthy individuals [26]. The study found that the mean serum neurogranin level in the infected group was increased compared to the control group.

In addition to investigating S100B, Grønhøj et al. evaluated neurofilament light chain (NFL) levels in adults with severe community-acquired acute BM and compared them to healthy controls [23]. Neurofilament light chain is a neuronal protein polymer that supports neuronal structure. Measurements were taken from the day of admission to the sixth day. The study revealed that serum NFL levels on day 1 were significantly higher than in the healthy controls, which may support its utility in BM diagnosis. Additionally, the same study assessed neuron-specific enolase (NSE), a marker expressed in neuronal cytoplasm widely discussed as an indicator of neuroinflammation. Researchers demonstrated a significant increase in NSE levels on the day of admission, which continued to rise until the third day. However, a visible decline in NSE levels was observed on day 4 compared to days 1 and 3. However, a separate study by Lins et al. found that NSE was not a significant diagnostic serum biomarker for any considered CNS infections (BM, VM, and neuroborreliosis) [24]. Taking those divergent results into consideration, further studies are needed.

Neuronal acetylcholinesterase (AChE), an enzyme distributed extracellularly in the brain, has also been investigated as a potential serum biomarker of CNS injury. Mader et al. compared blood samples from adult patients with various neurological disorders to healthy controls [27]. The patients were classified into 13 diagnostic groups based on their brain diseases, including bacterial and VM. The serum values showed wide variation, with many results falling below the detection limit. The researchers observed that AChE levels in both bacterial and VM patients were predominantly below the detection limit. In contrast, higher levels were recorded in patients with more chronic conditions. These low AChE levels in meningitis patients may correlate with the severity of blood-brain barrier impairment, further supporting its potential validity and specificity as a diagnostic blood biomarker for meningeal conditions.

Epigenetics has recently been explored as a promising avenue for improving CNS infection diagnosis. Pan et al. conducted a study comparing patients with TBM or VM to healthy controls [28]. The researchers identified 28 differentially expressed miRNAs between the TBM and VM groups, 11 between TBM and healthy individuals, and 21 between the VM and control group. Additionally, an independent trial involving TBM, VM patients, and healthy controls revealed significant differences in the expression levels of four miRNAs in TBM patients compared to VM and healthy controls. These miRNAs were identified as miR-126-3p, miR-130a-3p, miR-151a-3p, and miR-199a-5p. The diagnostic precision evaluation showed miR-126-3p demonstrated the highest accuracy in differentiating TBM from VM. Furthermore, the researchers showed that a combination of the four indicated miRNAs could effectively distinguish TBM from the VM group and the control group. Thus, the miRNA profiles of TBM and VM patients may significantly enhance diagnostic capabilities in differentiating between these two diseases.

Moreover, Olie et al. conducted a study analyzing 12 different inflammatory biomarkers in the serum and CSF of over seven hundred patients suffering from CNS infections (including BM, VM, and encephalitis) [29]. The biomarkers included CRP, PCT, C-X-C motif chemokine ligand (CXCL-10), macrophage-derived chemokine (MDC), interleukin 6 (IL-6), IL-8, IL-10, tumor necrosis factor-alpha (TNF-α), macrophage migration inhibitory factor (MIF), IL-1RA, CXCL13, and IL-1β. In distinguishing bacterial from viral CNS infections, CRP, PCR, CXCL13, and IL-6 showed moderate performance and procalcitonin demonstrated adequate diagnostic ability for BM. However, none of the serum biomarkers were precise enough (AUC  >  0.80) to reliably diagnose BM or VM. The statistical variability observed in the inflammatory blood biomarkers highlights the need for further research to validate their reliability in diagnosing meningitis. All the studies covered in this paragraph with additional information are summarized in Table 2.

### 3.3. Blood Procalcitonin and C-Reactive Protein Are Valuable in Pediatric Meningitis

Moving to the pediatric population, most of the included research focused on the biomarkers of PCT [30,31,32] and CRP [33,34] or both [35,36,37,38]. Some studies also considered other biomarker types [39] or models involving some of the mentioned markers [38]. Regarding the age of the evaluated population, some authors recruited neonates [31,32], while others also considered the much broader age spectrum.

Chaudhary et al. carried out a study comparing the serum concentration of PCT in populations with BM and non-BM [30]. They observed the PCT serum levels in BM to be markedly higher than those in non-BM. Moreover, the authors contrasted PCT performance with elevated serum total leukocyte count, showing a sensitivity of 81.8% but a low specificity of 50.0%. Dutta et al. examined the effectiveness of PCT plasma concentrations in diagnosing BM in infants with suspected sepsis, separately assessing the groups of definite and no definite meningitis [31]. The calculated cut-off values for both ‘definite’ and ‘definite or probable’ meningitis cannot be, however, credibly assessed due to the statistical insignificance. The authors concluded that plasma PCT cannot distinguish between the presence and lack of meningitis in neonates with clinical sepsis. The study of Rajial et al. included a comparison of serum PCT concentrations in three neonate groups: confirmed (positive CSF cytochemistry and culture), probable (positive CSF cytochemistry but negative culture), and non-meningitis (CSF cytochemistry and culture negative) [32]. A notable difference was found between all the groups. Furthermore, the researchers also examined CSF PCT and the ratio of serum to CSF PCT, which were demonstrated to have higher sensitivity and specificity in relation to serum PCT, and the first was commented as a more specific marker in the diagnosis of neonatal meningitis.

Sutinen et al. investigated the serum CRP values in the context of CNS infection differentiation [33]. In the case of the BM etiology, the lowest value of CRP in serum measured was 43 mg/L; all the remaining ones were over 50 mg/L, and the majority even over 100 mg/L. The authors highlighted the role of the normal values of serum CRP (defined as <10 mg/L) in ruling out BM. At the same time, they emphasized that the increased serum CRP value may also result from conditions such as TBM, brain abscess, and some viral infections observed in the reviewed group of patients. Pemde et al., on the other hand, examined a broader group of patients, including TBM, which was excluded or not considered in previous works [34]. In this study, the authors evaluated the CRP using a qualitative test recognizing CRP concentrations of 6 mg/mL or more. The authors designed three groups of patients: pyogenic meningitis or TBM and no meningitis. In the first two, 100% of the above-mentioned CRP tests were positive, whereas in the third, only 53%. This enabled them to point out negative serum CRP’s meaning in excluding BM.

Dubos et al. interpreted the results of six emergency departments across five different European countries examining blood levels of PCT, CRP, white blood cell count, and neutrophil count in patients with BM and aseptic meningitis [35]. Researchers found PCT concentration to be the best biomarker out of the investigated ones. CRP in serum was also described as having notable sensitivity and specificity; however, it had a much lower diagnostic value. The study of El Shorbagy et al. analyzed the serum levels of PCT, CRP, and leukocyte count in patients with clinical suspicion of meningitis, comparing BM to aseptic meningitis [36]. The authors found a positive correlation between the concentration of all three parameters, which turned out to be more significant in patients with positive bacterial cultures. It was, hence, reasoned for serum PCT to be of value as a diagnostic biomarker of acute BM. Moreover, it was also examined as valuable in evaluating the effects of antibiotic therapy. A study by Ibrahim et al. compared the values of PCT, CRP, and blood leukocyte count in patients with BM and non-BM [37]. The authors proved the values of serum PCT to be markedly higher in meningitis of bacterial etiology. Regarding CRP and blood leukocytes, the cut-off values differentiating the etiologies had lower sensitivity and specificity.

Babenko et al. carried out a study in which they tried to facilitate the differentiation between BM and VM of enteroviral etiology using a machine learning process [38]. Out of investigating the algorithm variables such as gender, age, clinical data, and laboratory results, they found PCT and CRP to have the best performance for BM diagnosis with a sensitivity of 100%, a specificity of 96%, and an accuracy of 98%. The authors also emphasized how the less time-consuming and minimally invasive compared to the standard CSF culture method of the proposed model may optimize the diagnostics of meningitis for clinicians. Gowin et al., on the contrary, analyzed data from hospitalizations of children with meningitis [39]. A broader spectrum of blood parameters included the concentrations of CRP, D-dimers, fibrinogen, glucose, and leukocytes. Those were juxtaposed between patients with aseptic and BM. C-reactive protein testing was found to have the highest sensitivity and specificity. Also, D-dimers and fibrinogen presented significant differences between patients with meningitis of both etiologies. All the studies covered in this paragraph with additional information are summarized in Table 3.

### 3.4. Other Blood Indicators of Pediatric Meningitis

Some of the studies apart from the CRP and PCT analyses also included additional markers and usually compared their effectiveness; therefore, we decided to describe them in a separate paragraph [40,41,42,43]. Moreover, other researchers investigated completely novel parameters without comparison to CRP or PCT [43,44,45,46].

Gao et al. analyzed the values of platelet to albumin (PAR) and lactate dehydrogenase to albumin (LAR) ratio in infants with suppurative meningitis, evaluating their role in identifying the refractory suppurative meningitis group [40]. Also, PCT and CPR were checked. The cut-off values were determined for all the markers, which include CRP, PCT, PAR, and LAR, with similar sensitivity (68%, 64%, 64%, and 68%, respectively) but more varied specificity (74.4%, 91.5%, 85.4%, and 86.6%, respectively). The authors also assessed the sensitivity and specificity of all four parameters combined, which both reached over 80%. Therefore, PAR and LAR were shown as a notable facilitation of the refractory suppurative meningitis diagnosis. Manyelo et al. explored the effectiveness of cytokine screening platforms, focusing specifically on the diagnosis of TBM [41]. The study involved the use of a modified version of adult pulmonary TB 7-marker signature (including CRP, interferon-gamma (IFN-γ), CXCL-10, complement factor H (CFH), apolipoprotein A1 (Apo-A1), serum amyloid A (SAA), and neural cell adhesion molecule 1 (NCAM1)) and a specific 3-marker biosignature (comprising adipsin, amyloid-beta 42 (Aβ42), and IL-10) in a population of children suspected to have meningitis. Interestingly, the latter presented even higher sensitivity and specificity. Furthermore, Sanaei Dashti et al. evaluated the blood parameters of erythrocyte sedimentation rate (ESR), CRP, ferritin, and PCT in children categorized into groups of BM and VM [42]. Serum CRP was demonstrated to have the highest efficacy in the context of BM diagnosis. Additionally, serum ESR was mentioned as a useful marker in the same context. Finally, Saleh et al. conducted an experiment in which they measured and compared the levels of PCT, CRP, ESR, total white blood cell (WBC) count, absolute neutrophil count (ANC), and neutrophil to lymphocyte ratio (NLR) in febrile children with seizure with suspected BM [43]. Researchers considered PCT to be of the most value in the diagnosis of BM juxtaposed to NLR and CRP.

Chen et al. investigated the plasma concentrations of B7-H3, TNF-α, IFN-γ, and IL-17 in pediatric patients with aseptic meningitis, BM, and non-meningitis [44]. The concentrations of B7-H3 and IL-17 were significantly higher in children with BM. The character of two other parameters, IFN-γ and IL-17, was evaluated not to be discriminatory regarding the meningitis diagnosis. The concentration of IFN-γ concededly was higher in groups with BM and aseptic meningitis than a control group; however, it did not show a significant difference between BM and aseptic meningitis. The levels of IL-17, in turn, were not elevated in either of the groups. Debray et al. conducted a retrospective bicentric analysis of eosinophilia among the following patient groups: documented BM, documented VM, and undocumented meningitis [45]. In the case of BM, the most effective diagnostic cut-off value for eosinophil count was set at <5/mm^3^. The authors have praised the role of eosinophilia as a meningitis biomarker; however, the possible better use of procalcitonin was emphasized. It was presumed that eosinophilia may be a valuable marker, especially in countries with low- and middle-income incomes, due to its cost-effectiveness.

Hou et al. investigated a group of neonates with suspected CNS infectious diseases using 2bRAD-M, an ultra-sensitive metagenomic sequencing method [46]. The authors demonstrated that in blood specimens of the study’s neonates, *Alloprevotella tannerae*, *Anoxybacillus A rupiensis*, *Brevundimonas vesicularis*, *Comamonas tsuruhatensis*, *Kocuria palustris*, *Massilia sp003484545*, *Pseudomonas E putida*, and *Massilia sp002354135* were meaningfully increased in the BM group, whereas *Cutibacterium acnes* was considerably elevated in the group without BM. This study showed the potential of microbiological biomarkers in terms of meningitis diagnosis. Finally, Mohamed et al. examined the role of a flow cytometric detection of CD64 surface marker on blood neutrophils in differentiating acute BM from patients without BM and healthy controls [47]. The expression of the CD64 marker on neutrophils in BM was markedly increased compared to non-BM and the control group. Therefore, the portrayed data indicate blood neutrophil CD64 expression as a relevant biomarker of bacterial infection. All the studies covered in this paragraph with additional information are summarized in Table 4.

### 3.5. Blood Biomarkers in Severe Bacterial Infections, Including Meningitis Among Children

Serious bacterial infections (SBIs) in children should be diagnosed at an early stage of development, as untreated cases can lead to severe complications. Serum apolipoprotein E (ApoE) is a glycoprotein primarily produced by hepatocytes, but it has also been already associated with neurological damage in the brain [48]. Fu et al. conducted a study in which the serum levels of ApoE were compared in pediatric patients [49]. The study sample included one group suffering from various bacterial infections, such as BM, and another group of healthy controls. The results demonstrated that the serum ApoE levels were significantly increased in the patients with bacterial infections but not aseptic meningitis compared to the healthy controls. Additionally, using a sepsis mouse model, the researchers assessed ApoE expression levels. Compared with the non-septic control group, plasma ApoE levels in the septic group increased during the early stages of infection at 1 h, 3 h, and 24 h. Moreover, in another study, Wang et al. compared the serum ApoE levels in pediatric patients with confirmed infections, including sepsis and bacterial and aseptic meningitis, to healthy controls [50]. The results showed that the serum ApoE levels were significantly higher in the patients with BM compared to those with aseptic meningitis or the control group. The data suggest a strong correlation between increased serum ApoE levels and invasive bacterial infections, such as BM, compared to non-invasive ones. This indicates that serum ApoE may enhance the diagnostic process in pediatric patients by distinguishing between bacterial and aseptic meningitis.

Carrol et al. [51] conducted a study investigating the diagnostic utility of five blood biomarkers in Malawian children with SBI. Two of these biomarkers, PCT and CRP, have already been widely discussed in this review. The researchers also evaluated soluble triggering receptors expressed on myeloid cells-1 (sTREM-1), a soluble form of TREM-1; hemoglobin scavenger receptor (CD163); and high mobility group box 1 (HMGB1). Serious bacterial infections included either BM or pneumonia and were compared to children without detectable disease. The researchers found that the CRP and PCT levels were significantly higher in the children with SBI compared to the healthy controls. No significant differences were observed in the median levels of CRP, CD163, or HMGB1 between the cases of BM and bacterial pneumonia. Interestingly, the median PCT levels were visibly elevated in BM, while the median sTREM-1 levels were decreased compared to pneumonia. Among the biomarkers, CRP and PCT emerged as the most accurate diagnostic indicators for SBI. A CD163 molecule, in contrast, was found to be a predictor of disease outcomes rather than a diagnostic biomarker, as a significant increase was observed in children who did not survive pneumococcal bacteremia. However, since the SBI cases analyzed in this study included both BM and pneumonia without distinguishing between them, the findings may lack specificity for diagnosing meningitis.

Neutrophil gelatinase-associated lipocalin (NGAL) is a siderophoric protein reported to be upregulated during inflammatory states via the NF-κB pathway. Resistin, a hormone secreted by adipose tissue, also increases in response to pro-inflammatory cytokines and NF-κB activation during SBI, such as sepsis or meningitis [52]. Irwin et al. investigated the levels of these two novel biomarkers in children with confirmed SBI, comparing them to children with non-bacterial infection and healthy controls [52]. The researchers found that the relative gene expression of resistin and NGAL was significantly increased in the SBI cases compared to the controls. Furthermore, the plasma concentrations of NGAL and resistin were significantly higher in the children with SBI compared to those with non-bacterial infections or healthy controls. Due to their high diagnostic accuracy, especially combined, NGAL and resistin are promising biomarkers that may enhance the diagnosis of SBI in children, including meningitis.

Ubenauf et al. conducted a study in which lipopolysaccharide-binding protein (LBP) levels were measured in children with either bacterial sepsis or meningitis and compared to a control group of uninfected children [53]. The median LBP serum concentrations at admission in patients with bacterial infections were significantly elevated compared to the control group. However, no significant difference was observed between the children with meningitis and those with invasive bloodstream infections. These findings suggest that elevated LBP serum concentrations can identify children with SBI, but they may lack the specificity to differentiate between meningitis and invasive bloodstream infections. All the studies covered in this paragraph with additional information are summarized in Table 5.

### 3.6. Blood Biomarkers of Meningitis in Patients of All Ages

Finally, some studies included patients of all ages, with both adult and pediatric populations [54,55,56,57,58,59]. Kandil et al. conducted a promising study about the potential role of serum and CSF heparin-binding protein (HBP) as a new marker in the prediction of acute BM and as a potential factor in differentiating BM from non-BM [54]. The studied population with clinical suspicion of acute meningitis was divided into three groups: patients diagnosed with acute BM, patients suffering from other types of meningitis, and subjects with normal CSF examination. Their work demonstrated a statistically significant difference in CSF HBP and serum HBP levels in the patients with acute BM compared to the two latter groups. Both parameters presented higher accuracy than the overall accuracy of CSF WBCs, CSF neutrophils, CSF protein, and CSF lactate. Another potential biomarker was analyzed in the study by Knudsen et al. [55]. The authors evaluated the diagnostic value of the serum levels of sCD163 and compared its efficacy with CRP and PCT in differing meningitis and bacterial infections. Patients were divided into two groups depending on suspicion of meningitis or another infectious disease. Further, subgroups were distinguished in both groups depending on the suspected etiology. The results indicated the superior role of CRP in terms of the diagnostic accuracy of bacterial infection, including meningitis, in comparison to sCD163. Despite that, sCD163 presented potential use as an additional marker for the rapid classification of patients suspected of meningitis.

Some studies, although they included blood biomarkers, showed an advantage of CSF examination in meningitis differentiation [56,57,58,59]. A study carried out by Kalchev et al. considered the use of pro- and anti-inflammatory cytokines such as interleukin 6 (IL-6), IL-8, IL-10, IL-12, TNF-α, classical CSF parameters (cell count, glucose and protein levels, and CSF/glucose ratio), and serum CRP as biomarkers in establishing the etiology of suspected acute CNS infection [56]. The authors found that the combined testing of CSF IL-12 and serum CRP is characterized by the highest diagnostic accuracy in terms of bacterial neuroinfection, including meningitis, followed by CSF IL-12 and CSF protein levels. This suggests the combination could be superior in discriminating between bacterial and viral acute infections of the CNS. However, most of the high-value biomarkers revealed in this study were detected in CSF. Mentis et al. aimed to investigate the utility of the NLR in the differential diagnosis of acute meningitis [57]. The authors analyzed the CSF and blood samples from over four thousand patients with a first episode of community-acquired meningitis. The results indicated that both NLR and neutrophil count yield the ability to discriminate between BM and VM; however, the effectiveness of those markers was significantly higher in CSF than in blood. Moreover, the authors stated that according to the performed logistic regression analysis, the joint presence of the NLR and neutrophil counts above the established cut-off values strongly suggest BM. Peng et al. conducted a study about the diagnostic value of delta-like 1 ligand (DLL1) in CSF and serum regarding TBM diagnosis [58]. TBM patients were compared to the following groups: VM/encephalitis, BM, intracranial metastatic tumor, and non-diagnosis, with the two last groups serving as the controls. The results showed the highest mean DLL1 CSF concentration in the TBM group, and the majority of those patients displayed levels > 1.0 ng/mL, with a highly statistically significant difference between the TBM group and the other groups where only one individual presented concentration > 1 ng/mL. In terms of serum DLL1 concentration, it was highest for the TBM and intracranial metastatic tumor groups, significantly differing from the remaining groups. However, no significant difference was found between the TBM and tumor groups. Nevertheless, those results indicate a promising role of DLL1 as a new biomarker in diagnosing TBM, however, more specifically in CSF. Finally, Zhang et al. analyzed the diagnostic accuracy of routine blood examinations and CSF lactate level in post-neurosurgical BM compared to post-neurosurgical aseptic meningitis [59]. This study involved data from over eight thousand patients who underwent neurosurgery. The authors found that routine blood examinations, including especially the WBC count, neutrophil proportion, platelet counts, and sodium concentration, had low diagnostic accuracy with AUC-ROC values < 0.7. However, the results of the CSF lactate level were much more promising, indicating its ability to distinguish aseptic meningitis from BM accurately. All the studies covered in this paragraph with additional information are summarized in Table 6.

## 4. Conclusions and Future Perspectives

This systematic review of the available literature presented the potential role of blood biomarkers in distinguishing different types of meningitis. The treatment of meningitis depends on the etiology of the disease, which is usually assessed by performing a lumbar puncture. Naturally, lumbar puncture can sometimes be contraindicated, for instance, due to impaired coagulation parameters. Moreover, some difficulties in performing can arise in specific cases. Finally, some patients may refuse consent for the procedure. In all these situations, the alternative method, which is not that invasive, would definitely ease meningitis management.

Blood biomarkers, such as the inflammatory markers CRP and PCT, are known to be altered in infectious diseases for a long time. Moreover, their specific levels can predict either bacterial or viral disease. The reviewed studies mark the diagnostic value of biomarkers like PCT and CRP in differentiating between bacterial and non-BM, both in adult and pediatric patients. There are certain differences among the found cut-off values and sensitivities; however, across the majority of the described studies, serum PCT seemed to show greater effectiveness and credibility than CRP. Moreover, in some studies, serum PCT seemed to be more useful than CSF PCT, increasing the role of blood testing in meningitis differentiation. Finally, emerging approaches like the presented machine learning model combining both markers within the interpretation show a very promising role of computed diagnostic tools.

**Figure 2:**
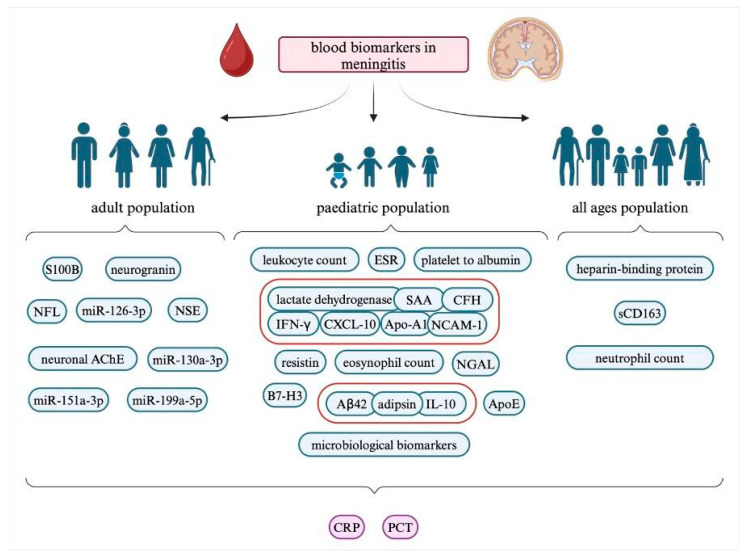
A summary of putative blood biomarkers in meningitis with a division to different age populations. Aβ42, amyloid-beta 42; AChE, acetylcholinesterase; Apo, apolipoprotein; CD, cluster of differentiation; CFH, complement factor H; CRP, C-reactive protein; CXCL-10, C-X-C motif chemokine ligand 10; ESR, erythrocyte sedimentation rate; IFN-γ, interferon-gamma; IL, interleukin; miR, microRNA; NCAM1, neural cell adhesion molecule 1; NFL, neurofilament light chain; NGAL, neutrophil gelatinase-associated lipocalin; NSE, neuron-specific enolase; PCT, procalcitonin; S100B, S100 calcium-binding protein B; SAA, serum amyloid A; sCD163, soluble CD163.

Apart from inflammatory markers, many others have been investigated by researchers. Figure 2 shows the analyzed biomarkers that have been mentioned in the review with an additional division based on the population. Molecules such as HBP or NLR could possibly serve as great individual serum biomarkers in the diagnostic process and differentiation of meningitis. HBP especially stands out with its excellent 100% sensitivity and specificity, both in serum and CSF. Although sCD163 offers a significantly lower diagnostic value than CRP or PCT, it could find a possible use in the rapid identification of patients with a systemic bacterial infection.

The presented diagnostic markers in the pediatric population beyond traditional CRP and PCT, such as PAR, LAR, and cytokine signatures, exhibit a potential contribution in identifying specific types of meningitis, however, with different sensitivities and specificities. Additionally, the presented novel biomarkers such as B7-H3, eosinophil count, CD64 neutrophil expression, or even a type of metagenomic sequencing method also convey hope in distinguishing BM from non-BM. Furthermore, while biomarkers such as S100B, NFL, and neurogranin show promise for diagnosing BM and other CNS infections, their diagnostic utility is often limited by variability and overlapping with other conditions. Serum biomarkers such as already mentioned CRP and PCT, or others, including ApoE, NGAL, and resistin, demonstrate significant potential for diagnosing serious bacterial infections in children. These biomarkers also show varying degrees of sensitivity and specificity, with ApoE and PCT particularly standing out for distinguishing bacterial meningitis from other conditions. While some markers, such as LBP and CD163, offer insight into disease outcomes or general inflammation, their specificity for meningitis remains limited. Collectively, these findings underscore the importance of integrating multiple biomarkers to enhance diagnostic accuracy for pediatric SBI. Finally, emerging tools like miRNA profiling demonstrate significant potential, particularly for distinguishing TBM from VM with high sensitivity and specificity. Regarding the recent Nobel Prize granted to Victor Ambros and Gary Ruvkun for their work on miRNA, their value seems even higher and should be further explored. Table 7 summarizes the sensitivity and specificity of the biomarkers analyzed in the review, highlighting the highest values. As can be seen, traditional and well-known inflammation markers, such as CRP and PCT, were shown to present the highest sensitivity and specificity with reasonable costs for conducting them. However, it should be noted that they are, especially CRP, markers that can be high in multiple conditions; thus, being even too sensitive can lead to false diagnoses. The summary presents that the tests that usually determine higher costs, for instance, by performing polymerase chain reaction (PCR), may have lower specificity and sensitivity in diagnosing meningitis. Sometimes, providing real-time PCR can be sufficient for meningitis diagnosis, especially in cases when the inflammation is systemic. Nevertheless, the cost-effectiveness of using simple diagnostic tools such as CRP and PCT seems to be the highest. Moreover, a promising alternative for lumbar puncture can be the combined measurements of CRP and PCT together. The costs are clearly the advantage of that solution compared to including in the diagnostic process other, more complicated biomarker testing. Importantly, although blood biomarkers are a promising addition to the typical meningitis diagnostic tools, it should be remembered that in a lot of cases, they can be insufficient. Therefore, clinicians should be very careful to avoid potential mistakes. Being not invasive is an undeniable advantage; however, the big disadvantage may be missing the proper diagnosis, and physicians should be aware of it. After all, making the correct diagnosis, followed by implementing the right therapy, is the most important part of meningitis management.

While the described findings expand the diagnostic toolkit in the area of meningitis, many of them require further validation in additional studies due to the limited sample sizes or the retrospective character of the study designs. Further studies are necessary to ensure reliable and precise diagnostic strategies for CNS infections. However, it is highly possible that in the near future, some blood biomarkers may not replace but will facilitate the meningitis diagnosis.

## 5. Limitations

Undoubtedly, one of the limitations of this review, as well as the particular studies that have been cited, is the small number of participants, which can influence the significance and value of the obtained results. Moreover, the analyzed studies mainly focused on distinguishing between bacterial and viral or aseptic meningitis. Therefore, the lacking point is taking into consideration other types of meningitis, such as TBM. Finally, much research regards simple inflammatory markers, so the number of studies investigating other types of biomarkers is limited. According to the methodology of the systematic review, the consideration of articles only in English may lead to omitting valuable research written in another language. Moreover, using only specific etiologies of meningitis in the search strategy could potentially lead to missing an article in which the etiology is different or not specified.

## Figures and Tables

**Table 1:** A summary of studies regarding PCT and CRP in adult meningitis.

Ref.	Year	Population	Comparison	Biomarker	Findings	Sensitivity and Specificity	Possible Use
Shen et al. [16]	2015	45 adult BM pts	75 non-BM adult pts	PCT	↑ serum and CSF PCT in BM pts; ↑ serum PCT AUC for BM (0.96) than CSF PCT AUC (0.90)	87% and 100% (serum PCT, cut-off 0.88 ng/mL)	both serum and CSF PCT as BM diagnostic biomarkers
Viallon et al. [17]	2011	35 adult BM pts	181 VM adult pts	PCT	the highest value in BM diagnosis had CSF lactate and serum PCT	94% and 92% (serum PCT, cut-off 0.28 ng/mL)	serum PCT as BM diagnostic biomarker
Zhang et al. [18]	2017	24 suppurative meningitis adult pts, 20 adult VM pts, and 22 adult TBM pts	20 control pts	serum PCT	↑ serum PCT in suppurative meningitis pts than in others	-	serum PCT as suppurative meningitis diagnostic biomarker
Karan et al. [19]	2024	30 adult BM pts	52 non-BM pts	serum PCT	NSD between CSF and serum PCT sensitivity and specificity in BM diagnosis	83.3% and 86.5% (serum PCT, cut-off 0.6 ng/mL)	serum PCT as BM diagnostic biomarker
Alnomasy et al. [20]	2021	38 adult BM pts	34 VM pts and 3 mixed infection pts	CRP, PCT, blood and CSF LEU, CSF-protein, CSF-glucose	↑ PCT, blood and CSF-LEU, and CSF-protein; ↓ CSF-glucose in BM than VM pts	100% and 97.1% (PCT and CSF-protein combined)	PCT with an additional value to CSF testing in BM diagnosis
Takada et al. [21]	2024	27 adult AM pts and 1 adult BM pts	52 non-meningitis pts	CRP	↑ CRP in BM pts (21.7 mg/dL) compared to non-meningitis pts (5.6 mg/dL) and AM pts (0.2 mg/dL)		CRP as an additional BM diagnostic biomarker
Morales Casado et al. [22]	2016	38 BM pts (>15 years old)	33 VM pts, 15 probable VM pts, and 12 partially treated acute meningitis pts	PCT, CRP	↑ PCT in BM (11.47 ng/m) than in VM (0.10 ng/mL)	67.5% and 86.3% (CRP) 94.6% and 72.4% (PCT)	PCT as an acute meningitis diagnostic biomarker

↑, increase; ↓, decreased; AUC, area under curve; BM, bacterial meningitis; CRP, C-reactive protein; CSF, cerebrospinal fluid; LEU, leukocyte count; NSD, no statistical difference; PCT, procalcitonin; pts, patients; Ref., reference; TBM, tuberculous meningitis; VM, viral meningitis.

**Table 2:** A summary of studies regarding blood biomarkers other than PCT and CRP in adult meningitis.

Ref.	Year	Population	Comparison	Biomarker	Findings	Sensitivity, Specificity	Possible Use
Grønhøj et al. [23]	2021	15 adult pts with severe community-acquired acute BM	HC	NFL, NSE, and S100B	serum NFL ↑ from day 1 up to day 3–6, peaking day 6; serum NSE ↑ from admission up to day 3; the highest median serum S100B value at admission (0.10 mg/L)	-	NFL, NSE, and S100B as acute BM diagnostic biomarkers
Lins et al. [24]	2005	32 adult pts (11 BM, 13 VM, and 8 meningoencephalitis or neuroboreliosis)	13 noninfectious CNS disease or SAH pts	S100B and NSE	the highest S-100B values on admission; S100B ↑ in BM than others; NSD in NSE levels	-	S100B as a BM diagnostic biomarker
Unden et al. [25]	2004	57 pts (aged 15 to 84 years)	various CNS infections, including BM and VM	S100B	↑ serum S100B > 0.15 mg/L in 33% pts; ↑ S100B in 73% BM pts compared to 7% VM pts	-	S100B as a BM and VM diagnostic biomarker
Canturk et al. [26]	2022	55 adult meningitis pts	15 HC	neurogranin	↑ serum neurogranin in meningitis (429.2 ng/mL) than in HC (198.6 ng/mL)	73% and 82%	serum neurogranin in meningitis diagnosis
Mader et al. [27]	1991	272 pts (aged 17 to 78 years) with neurological disorders, including BM, VM, and CID	9 HC	AChE	↓ AChE in BM and VM pts than in HC; ↑ AChE in CID pts	-	AChE as a BM and VM biomarker
Pan et al. [28]	2019	47 adult TBM pts and 44 adult VM pts	53 HC	miRNA	miR-126-3p, miR-130a-3p, miR-151a-3p, and miR-199a-5p efficient in distinguishing TBM from VM and HC; miR-126-3p with the highest accuracy in differentiating TBM from VM	81.8% and 90.0% for TBM vs. VM; 81.8% and 84.6% for TBM vs. non-TBM (diagnostic panel)	miRNAs as a TBM diagnostic biomarkers
Olie et al. [29]	2024	738 pts ≥16 years with various CNS infections, including 107 VM and 81 BM pts	VM and BM vs. other CNS infections	CRP, PCT, CXCL-10, MDC, IL-6, IL-8, IL-10, TNF-α, MIF, IL-1RA, CXCL13, and IL-1β	AUC values of CRP, PCT, CXCL13, and IL-6 between 0.70 and 0.80; PCT with adequate diagnostic ability for BM (AUC of 0.71)	-	diagnostic biomarkers of BM and VM

↑, increased; ↓, decreased; AChE, acetylcholinesterase; AUC, area under the curve; BM, bacterial meningitis, CID, chronic inflammatory diseases; CNS; central nervous system, CRP, C-reactive protein; CXCL, C-X-C motif chemokine ligand; HC, healthy controls; IL, interleukin; IL-1RA, interleukin-1 receptor antagonist; MDC, macrophage-derived chemokine; MIF, macrophage migration inhibitory factor; miRNA, microRNA; NFL, neurofilament light chain; NSD, no significant differences; NSE, neuron-specific enolase; PCT, procalcitonin; pts, patients; Ref., reference; RNA, ribonucleic acid; S100B, S100 calcium-binding protein B; SAH, subarachnoid hemorrhage; TBM, tuberculous meningitis; TNF-α, tumor necrosis factor-alpha; VM, viral meningitis; vs., versus.

**Table 3:** A summary of studies regarding PCT and CRP in pediatric meningitis.

Ref.	Year	Population	Comparison	Biomarker	Findings	Sensitivity, Specificity	Possible Use
Chaudhary et al. [30]	2018	22 BM pts (aged 3 months to 15 years)	28 NBM	serum PCT	↑ serum PCT in BM compared to NBM	95.45% and 84.6% (cut-off > 0.5 ng/mL)	serum BM diagnostic biomarkers
Dutta et al. [31]	2022	18 pts with ‘definite’ and 37 with ‘definite or probable’ meningitis	comparison within the group of 216 pts with suspected sepsis (aged 0–56 days)	serum PCT	cut-off values of >4.87 ng/mL in ‘definite’ and >0.425 ng/mL in ‘definite or probable’ meningitis	-	caution with the use of serum PCT in infants with clinical sepsis
Rajial et al. [32]	2022	17 neonates with confirmed and 25 with probable meningitis	25 non-meningitis pts	serum PCT	↑ serum PCT in meningitis compared to non-meningitis pts	92.9% and 76% (cut-off ≥ 1.38 ng/mL)	CSF PCT and ‘serum to CSF PCT’ ratio as meningitis biomarkers
Sutinen et al. [33]	1998	19 BM pts, 4 TBM pts, and 38 VM pts	comparison within 103 CNS infections pts (aged 0 months to >16 years)	serum CRP	↑ CRP in BM than in VM	94% and 65% (cut-off > 50 mg/mL)	role of normal values of serum CRP (defined as <10 mg/L) in excluding BM
Pemde et al. [34]	1996	40 TBM pts and 30 pyogenic meningitis (aged 1 month to 12 years)	40 non-meningitis pts	serum CRP (qualitative test (+) > 6 mg/mL)	100% positive CRP tests in study groups and 53% positive in controls	-	role of negative serum CRP in excluding BM
Dubos et al. [35]	2008	96 ED BM pts (aged 29 days to 18 years)	102 AM pts	serum PCT, serum CRP, WBC count, and neutrophil count	↑ serum PCT in BM than in AM (the most specific marker); CRP able to differentiate BM and AM with a cut-off at ≥20	99% and 83% (PCT, >0.5 ng/mL)	serum PCT as BM diagnostic biomarker in ED
El Shorbagy et al. [36]	2018	24 BM pts (aged 4 months to 14 years)	16 AM pts	serum levels of PCT, CRP, and leukocyte count	positive correlation between all 3 parameters	100% and 63% (PCT > 2 ng/mL); 86% and 82%(PCT > 10 ng/mL); 89% and 60%(CRP > 10 mg/dL); 74% and 78%(CRP > 20 mg/dL)	serum PCT as an acute BM diagnostic biomarker
Ibrahim et al. [37]	2011	18 BM pts (aged 2 months to 10 years)	20 NBM pts	serum PCT, CRP, and TLC	↑ PCT, CRP, and TLC in BM pts than in NBM pts	95% and 94% (PCT > 0.5 ng/mL); 80% and 90% (CRP > 10 mg/L); 70% and 66% (TLC < 4 or >15 × 10^9^/L)	serum PCT as the most specific BM biomarker
Babenko et al. [38]	2021	123 BM pts (aged 1 month to 17 years)	146 EVM	serum PCT and CRP	cut-offs calculated in ML process: PCT > 0.16 ng/mL (BM); CRP ≤ 31.2 mg/L (EVM)	100% and 96% for both markers used together	a role of ML in meningitis differential diagnosis
Gowin et al. [39]	2016	65 BM pts (aged 1 month to 18 years)	64 AM pts	serum CRP, D-dimers, fibrinogen, glucose, and leukocyte	↑ CRP (the most sensitive), D-dimers (>970 µg/L), and fibrinogen (>4.4 g/L) in BM compared to AM pts	98.46% and 100% (CRP > 84 mg/L)	BMS (by Nigrovic) and its components plausible in the BM diagnosis

↑, increase; AM, aseptic meningitis; BM, bacterial meningitis; BMS, bacterial meningitis score; CNS, central nervous system; CRP, C-reactive protein; CSF, cerebrospinal fluid; ED, emergency department; EVM, enteroviral meningitis; ML, machine learning; NBM, non-bacterial meningitis; PCT, procalcitonin; pts, patients; Ref., reference; TBM, tuberculous meningitis; TLC, total leukocyte count; WBC, white blood cell.

**Table 4:** A summary of studies regarding blood biomarkers other than PCT and CRP in pediatric meningitis.

Ref.	Year	Population	Comparison	Biomarker	Findings	Sensitivity, Specificity	Possible Use
Gao et al. [40]	2024	25 refractory suppurative meningitis pts (aged 0–1 years)	82 common suppurative meningitis	serum PCT, CRP, PAR, and LAR	PCT, CRP, and LAR ↑ in refractory than in common suppurative meningitis; PAR ↓ in refractory than in common suppurative meningitis	64% and 91.5% (PCT > 20.995 ng/mL); 68% and 74.4% (CRP > 86.185 mg/L); 64% and 85.4% (PAR < 10^9^/g); 68% and 86.6% (LAR 11.760 IU/L); 84% and 80.5% (all four together)	PAR and LAR as a biomarker of refractory suppurative meningitis
Manyelo et al. [41]	2019	47 pts (aged 3 months to 13 years)	effectiveness of screening platforms (specifically for TBM diagnosis)	TB 7-marker signature (CRP, IFN-γ, IP-10, CFH, Apo-A1, SAA, and NCAM1) and specific 3-marker signature (adipsin, Aβ42, and IL-10)	better performance of the 3-marker signature than the 7-marker signature	73.9% and 66.7% for the 7-marker signature; 82.6% and 75.0% for 3-marker signature	combination of biomarkers to facilitate TBM diagnosis
Sanaei Dashti et al. [42]	2017	12 BM pts (aged 28 days to 14 years)	38 VM	ESR, serum CRP, ferritin, and PCT	↑ ESR, serum CRP, and ferritin in BM than VM pts; NSD in PCT between BM and VM pts	85.86% and 67.87% (ESR > 30 mm/h); 91.16% and 100% (CRP > 57 mg/L); 81.1% and 62.9% (ferritin 47.3 ng/mL); 66.7% and 59.3% (PCT > 60 ng/dL)	low values in BM diagnosis of the investigated biomarkers
Saleh et al. [43]	2020	50 BM pts (aged 6 months to 5 years)	40 HC	serum PCT, CRP, ESR total WBC count, ANC, and NLR	↑ ANC, CRP, NLR, and PCT in BM pts than HC	72% and 100% (ANC > 8600 (×10^3^/μL)); 56% and 90% (CRP > 15 (mg/L); 52% and 100% (NLR > 2.5); 85% and 100% (PCT > 5.5 pg/mL)	PCT as the most valuable BM diagnostic biomarker
Chen et al. [44]	2009	6 BM pts and 16 AM pts (aged 2 months to 12 years)	12 non-meningitis pts	plasma B7-H3, TNFα, IFN-γ, and IL-17	↑ B7-H3 in BM (98.79 pg/mL) than in AM (32.37 pg/mL); ↑ TNF-α level in BM (198.18 pg/mL) than in AM (23.87 pg/mL); NSD for IFN-γ and IL-17	-	B7-H3 as a biomarker in BM and AM differential diagnosis
Debray et al. [45]	2019	45 BM pts and 73 VM pts (aged 3 months to15 years)	33 undocumented meningitis pts	eosinophil count	eosinophil count ↓ in BM pts than in other groups	80% and 73% (<5/mm^3^); 87% and 44% (<100/mm^3^)	a role of eosinophil count in BM diagnosis
Hou et al. [46]	2023	12 BM neonates	11 NBM neonates	microbial species	8 species ↑ in blood in BM than NBM (*Alloprevotella tannerae*, *Anoxybacillus A rupiensis*, *Brevundimonas vesicularis*, *Comamonas* *tsuruhatensis*, *Kocuria palustris*, *Massilia sp003484545*, *Pseudomonas* *Eputida*, and *Massilia sp002354135*); 1 species ↑ in blood in NBM than BM (*Cutibacterium acne*)	-	a value of microbiological biomarkers in BM diagnosis
Mohamed et al. [47]	2012	44 BM pts (aged 2 months to 11 years)	88 NBM pts	CD64 surface marker on blood neutrophils	↑ expression in BM (71.38%) compared to culture-negative meningitis (48.63%) and controls (4.37%)	100% and 65.1%	blood neutrophil CD64 as a BM diagnostic biomarker

↑, increased; ↓, decreased; AM, aseptic meningitis; ANC, absolute neutrophil count; Apo-A1, apolipoprotein A1; Aβ42, amyloid beta 42; B7-H3, CD276; BM, bacterial meningitis; CD, cluster of differentiation; CFH, complement factor H; CRP, C-reactive protein; ESR, erythrocyte sedimentation rate; HC, healthy controls; IFN-γ, interferon-gamma; IL, interleukin; IP-10, interferon gamma-induced protein 10; LAR, lactate dehydrogenase to albumin ratio; NBM, non-bacterial meningitis; NCAM1, neural cell adhesion molecule 1; NLR, neutrophil to lymphocyte ratio; NSD, no statistical difference; PAR, platelet to albumin ratio; PCT, procalcitonin; pts, patients; Ref., reference; SAA, serum amyloid A; TB, tuberculosis; TBM, tuberculous meningitis; TNFα, tumor necrosis factor-alpha; VM, viral meningitis; WBC, white blood cell.

**Table 5:** A summary of studies regarding blood biomarkers in severe bacterial infections, including meningitis in the pediatric population.

Ref.	Year	Population	Comparison	Biomarker	Findings	Sensitivity, Specificity	Possible Use
Fu et al. [49]	2014	279 bacterial infections pts (aged 0 to 6 years); a mouse sepsis model	58 HC; non-septic mice controls	serum ApoE	serum ApoE ↑ in BM (5.07 mg/dL) and ↓ in AM (3.62 ± 0.97 mg/dL) than in HC (3.68 mg/dL); ↑ plasma ApoE in septic mice vs. controls at 1 h postinoculation (1.32 mg/dL vs. 1.06 mg/dL), at 3 h (1.62 mg/dL vs. 1.04 mg/dL), and at 24 h (2.20 mg/dL vs. 1.12 mg/dL)	-	serum ApoE as a BM diagnostic biomarker
Wang et al. [50]	2011	94 infection pts including meningitis and sepsis (aged 1 month to 13 years)	91 non-confirmed infection pts	ApoE	↑ serum ApoE levels in sepsis and BM group than in AM and controls	85% and 100% (cut-off >1.7 mg/L)	ApoE as a BM diagnostic marker
Carrol et al. [51]	2009	282 pts with BM symptoms and 95 pts with pneumonia symptoms (aged 2 months to 16 years)	15 HC	CRP, PCT, sTREM-1, CD163, and HMGB1	↑ PCT in BM (44 ng/mL) compared to pneumonia (13 ng/mL); ↓ s-TREM-1 in BM (50 ng/mL) than in pneumonia (64 ng/mL); ↑ CRP and PCT in SBI (291, 46 ng/mL) than in HC (135, 3 ng/mL); NSD in CRP, CD163, or HMGB1 between BM and pneumonia; correlation between the studied biomarkers and IL-6, IL-8, IL-10, and IL-Ra	-	role as indicators of systemic bacterial infections, including BM
Irwin et al. [52]	2012	282 pts with BM symptoms and 95 pts with pneumonia symptoms (aged 2 months to 16 years)	15 HC	NGAL and resistin	↑ resistin and NGAL gene expression in SBI cases compared to HC; plasma NGAL and resistin ↑ in SBI than in NBI and HC (287 vs. 128 vs. 62 ng/mL and 195 vs. 90 vs. 18 ng/mL, respectively); AUC of 0.79 for NGAL, 0.80 for resistin, and 0.90 for NGAL, resistin, and PCT combined	86.7% and 50% (NGAL); 91.4% and 51.9% (resistin),	NGAL and resistin as diagnostic biomarkers of SBI
Ubenauf et al. [53]	2007	19 bacterial sepsis pts and 20 BM pts (aged 2 months to 17 years)	60 controls	LBP	↑ serum LBP at admission in the study group (45.0 ug/mL) vs. controls (8.3 ug/mL); NSD between BM and sepsis	97% and 77%	serum LBP as a bacterial infection diagnostic biomarker

↑, increased; ↓, decreased; AM, aseptic meningitis; ApoE, apolipoprotein E; AUC, area under the curve; BM, bacterial meningitis; CD, cluster of differentiation; CRP, C-reactive protein; HC, healthy controls; HMGB1, high mobility group box 1 protein; IL, interleukin; IL-Ra, interleukin receptor antagonist; LBP, lipopolysaccharide binding protein; NBI, no-detectable bacterial infection; NGAL, neutrophil gelatinase-associated lipocalin; NSD, no significant difference; PCT, procalcitonin; pts, patients; Ref., reference; SBI, serious bacterial infection; sTREM-1, soluble triggering receptor expressed on myeloid cell-1; vs., versus.

**Table 6:** A summary of studies regarding blood biomarkers of meningitis in patients of all ages.

Ref.	Year	Population	Comparison	Biomarker	Findings	Sensitivity, Specificity	Possible Use
Kandil et al. [54]	2018	30 acute BM pts and 30 VM pts (aged 1 to 50 years)	30 HC	serum and CSF HBP	↑ CSF HBP in BM (192.2 ng/mL) than in VM (3.3 ng/mL) and HC (0.82 ng/mL); ↑ serum HBP in BM (192.2 ng/mL) than in VM (3.7 ng/mL) and HC (0.84 ng/mL)	100% and 100%	HBP as a BML diagnostic biomarker even in partially treated cases
Knudsen et al. [55]	2007	55 pts (aged 12 to 92 years)	two divisions, one into purulent, serous, or non-meningitis, and the second into systemic, local, or non-bacterial disease	sCD163, CRP, and PCT	↑ serum sCD163 is the best in distinguishing bacterial from non-bacterial disease; ↓ sCD163 diagnostic accuracy (AUC = 0.72) than CRP (AUC = 0.91) and PCT (AUC= 0.87); AUCs < 0.75 for sCD163, CRP, and PCT in differentiating purulent meningitis from others	91% and 47% (sCD163)	sCD163 as an additional biomarker of bacterial infections
Kalchev et al. [56]	2021	21 BN pts, 14 VN pts, and 32 UN pts (aged 1 month to 88 years)	13 HC	IL-6, IL-8, IL-10, IL-12(p40), TNF-α cytokines, classical CSF parameters, and serum CRP levels	↑ CRP AUC from 0.943 to 0.995 when combining the CSF IL-12 and serum CRP in BN diagnosis	100% and 90.9% (cut-off 144)	combined CSF IL-12 and serum CRP as a biomarker panel of acute BN
Mentis et al. [57]	2016	4339 patients (aged 0 to 100 years)	BM vs. VM pts	NLR and NC	↑ NLR and NC in BM than VM pts; ↑ sensitivity, NPV, OR, and RR in CSF than blood for NLR and NC	84.8% and 79.6% (CSF NLR > 2) 46.4% and 83.0% (blood NLR > 8) 78.2% and 90.2% (CSF NC > 287) 51.9% and 81.0% (blood NC > 12,100)	NLR as a biomarker in meningitis differential diagnosis
Peng et al. [58]	2014	62 TBM pts, 38 VM/viral encephalitis pts, 26 BM pts, and 17 TUM pts (aged 2 to 74 years)	30 pts with no diagnosis	delta-like 1 ligand (DLL1)	↑ CSF DLL1 concentration in TBM (4.12 ng/mL with 87% >1.0 ng/mL) than in other pts; NSD in serum DLL1 between TBM and TUM pts, ↑ than in other groups	87% and 99% (CSF DLL1 > 1 ng/mL) 82% and 91% (serum DLL1 > 6 ng/mL)	DLL1 as a TBM diagnostic biomarker
Zhang et al. [59]	2017	554 post-neurosurgical BM pts (aged <14 to >60 years)	868 post-neurosurgical AM pts	routine blood examinations and CSF lactate	↑ CSF lactate in BM than AM; low diagnostic accuracy for routine blood examinations	76.36% and 87.79% (CSF lactate > 3.6 mmol/L)	CSF lactate as BM vs. AM biomarkers

↑, increased; ↓, decreased; AM, aseptic meningitis; AUC, area under the curve; BM, bacterial meningitis; BN, bacterial neuroinfection; CD, cluster of differentiation; CRP, C-reactive protein; CSF, cerebrospinal fluid; DLL1, delta-like 1 ligand; HBP, heparin-binding protein; HC, healthy controls; IL, interleukin; NC, neutrophil counts; NLR, neutrophil-to-lymphocyte ratio; NPV, negative predictive value; NSD, no significant difference; OR, odds ratio; PCT, procalcitonin; pts, patients; RR, relative risk; sCD163, soluble CD163; TBM, tuberculous meningitis; TNF-α, tumor necrosis factor-α; TUM, intracranial metastatic tumor; UN, unidentified neuroinfection; VM, viral meningitis; VN, viral neuroinfection; vs., versus.

**Table 7:** A summary of the sensitivity and specificity of the blood biomarkers with information about potentially diagnosed etiology and age group. The biomarkers with the highest values are additionally highlighted (sensitivity > 90%, blue; specificity > 90%, orange; both values > 90%, green).

Biomarker	Study	Study Design	Sensitivity	Specificity	Cut-Off	Meningitis Type	Age
PCT	[16]	prospective	87%	100%	>0.88 ng/mL	bacterial vs. non-bacterial	adults
PCT	[17]	prospective	94%	92%	>0.28 ng/mL	bacterial vs. viral	adults
PCT	[19]	cross-sectional	83.3%	86.5%	>0.6 ng/mL	bacterial vs. non-bacterial	adults
PCT	[22]	prospective	94.6%	72.4%	≥1.1 ng/mL	bacterial vs. viral	adults
CRP			67.5%	86.3%	≥90 mg/L		
neurogranin	[26]	prospective	73%	82%	>204.26 ng/mL	meningitis vs. non-meningitis	adults
microRNA panel	[28]	prospective	81.8%	90%	-	tuberculous vs. viral	neonates
			81.8%	84.6%	-	tuberculous vs. non-tuberculous	
PCT	[30]	cross-sectional	95.45%	84.6%	>0.5 ng/mL	bacterial vs. non-bacterial	neonates
PCT	[32]	prospective	92.9%	76%	≥1.38 ng/mL	meningitis vs. non-meningitis	children
CRP	[33]	prospective	94%	65%	>50 mg/mL	bacterial vs. viral	children
PCT	[35]	retrospective (secondary analysis)	99%	83%	>0.5 ng/mL	bacterial vs. aseptic	children
PCT	[36]	prospective	100%	63%	>2 ng/mL	bacterial vs. aseptic	children
PCT			86%	82%	>10 ng/mL		
CRP			89%	60%	>10 mg/dL		
CRP			74%	78%	>20 mg/dL		
PCT	[37]	prospective	95%	94%	>0.16 ng/mL	bacterial vs. non-bacterial	children
CRP			80%	90%	>10 mg/dL		
leukocytes			70%	66%	<4 or >15 × 10^9^/L		
PCT + CRP	[38]	prospective	100%	96%	PCT > 0.16 ng/mL CRP > 31.2 mg/L	bacterial vs. enteroviral	children
CRP	[39]	retrospective	98.46%	100%	>84 mg/dL	bacterial vs. aseptic	children
PCT	[40]	retrospective	61%	94.5%	>20.995 ng/mL	refractory suppurative vs. common suppurative	neonates
CRP			68%	74.4%	>86.185 mg/L		
LAR			64%	85.4%	<10^9^/g		
PAR			64%	86.6%	11.760 IU/L		
PCT + CRP + LAR + PAR			84%	80.5%	as above		
7-marker signature	[41]	prospective	73.9%	66.7%	-	tuberculous vs. others	children
3-marker signature			82.6%	75%	-		
ESR	[42]	cross-sectional	85.86%	67.87%	>30 mm/h	bacterial vs. viral	children
CRP			91.16%	100%	>57 mg/L		
Ferritin			81.1%	62.9%	47.3 ng/mL		
PCT			66.7%	59.3%	>60 ng/dL		
neutrophiles	[43]	not explicitly stated	72%	100%	>8600 × 10^3^/μL	bacterial vs. healthy	children
CRP			56%	90%	>15 mg/L		
NLR			52%	100%	>2.5		
PCT			85%	100%	>5.5 pg/mL		
eosinophils	[45]	retrospective	80%	73%	>5/mm^3^	bacterial vs. others	children
			87%	44%	>100/mm^3^		
CD64	[47]	prospective	100%	65.1%		bacterial vs. non-bacterial	children
ApoE	[50]	prospective	85%	100%	>1.7 mg/L	bacterial vs. others	children
NGAL	[52]	prospective	86.7%	50%	>100 ng/mL	bacterial infections vs. others	children
resistin			91.4%	51.9%	>80 ng/mL		
LBP	[53]	prospective	97%	77%	>13 μg/mL	bacterial infections vs. others	children
HBP	[54]	prospective	100%	100%	>45.3 ng/mL	bacterial vs. healthy	all
sCD163	[55]	prospective	91%	47%	>3.95 mg/L	bacterial infections vs. others	all
NLR	[57]	retrospective	46.4%	83%	>8	bacterial vs. viral	all
neutrophiles			51.9%	81%	>12,100		
DLL1	[58]	prospective	82%	91%	>6 ng/mL	tuberculous vs. others	all

ApoE, apolipoprotein E; CD, cluster of differentiation; CRP, C-reactive protein; DLL1, delta-like 1 ligand; ESR, erythrocyte sedimentation rate; HBP, heparin-binding protein; LAR, lactate dehydrogenase to albumin ratio; LBP, lipopolysaccharide-binding protein; NGAL, neutrophil gelatinase-associated lipocalin; NLR, neutrophil to lymphocyte ratio; PAR, platelet to albumin; PCT, procalcitonin; s, soluble.

## Data Availability

Not applicable.

## Supplementary Materials

The supporting information can be downloaded at: https://www.mdpi.com/article/10.3390/ijms26041427/s1.

## Abbreviations

The following abbreviations are used in this manuscript:

Aβ42	amyloid-beta 42
AChE	acetylcholinesterase
ANC	absolute neutrophil count
Apo	apolipoprotein
AUC	area under curve
BM	bacterial meningitis
CD	cluster of differentiation
CFH	complement factor H
CNS	central nervous system
CRP	C-reactive protein
CSF	cerebrospinal fluid
CXCL	C-X-C motif chemokine ligand
DLL1	delta-like 1 ligand
ESR	erythrocyte sedimentation rate
HBP	heparin-binding protein
HMGB1	high mobility group box 1
HSV	Herpes simplex viruses
IFN-γ	interferone gamma
IL	interleukin
LAR	lactate dehydrogenase to albumin ratio
LBP	lipopolysaccharide-binding protein
MDC	macrophage-derived chemokine
MIF	macrophage migration inhibitory factor
miR	microRNA
NCAM1	neural cell adhesion molecule 1
NFL	neurofilament light chain
NGAL	neutrophil gelatinase-associated lipocalin
NLR	neutrophil to lymphocyte ratio
NSE	neuron-specific enolase
PAR	platelet to albumin ratio
PCT	procalcitonin
PRISMA	Preferred Reporting Items for Systematic Reviews and Meta-Analyses
S100B	S100 calcium-binding protein B
SAA	serum amyloid A
SBI	serious bacterial infections
sTREM-1	soluble triggering receptors expressed on myeloid cells-1
TBM	tuberculous meningitis
TNF-α	tumor necrosis factor-alpha
VM	viral meningitis
WBC	white blood cell
WHO	World Health Organization
