# Supplementary material for: Investigating Blood Biomarkers That Can Facilitate the Diagnosis of Meningitis—A Systematic Literature Review
Source: Int J Mol Sci. 2025 Feb 8;26(4):1427. doi: 10.3390/ijms26041427 (PMC11855063; doi:10.3390/ijms26041427)
Supplement: Supplementary file 1 [file ijms-26-01427-s001.zip › ijms-3454673-supplementary.pdf]

Supplementary material Table S1. Quality assessment of the included studies using the methodological quality appraisal tool [15].

Ref.	Year	C1.	C2.	C3.	C4.	C5.	Total
Shen *et al.* [16]	2015	1	1	1	1	1	5
Viallon *et al.* [17]	2011	1	1	1	1	1	5
Zhang *et al.* [18]	2017	1	1	1	1	1	5
Karan *et al.* [19]	2024	1	1	1	1	1	5
Alnomasy *et al.* [20]	2021	1	1	1	1	1	5
Takada *et al.* [21]	2024	1	1	1	1	1	5
Morales Casado *et al.* [22]	2016	1	1	1	1	1	5
Grønhøj *et al.* [23]	2021	1	1	0	1	1	4
Lins *et al.* [24]	2005	1	1	1	1	1	5
Unden *et al.* [25]	2004	1	1	1	1	1	5
Canturk *et al.* [26]	2022	1	1	1	1	1	5
Mader *et al.* [27]	1991	1	1	1	1	1	5
Pan *et al.* [28]	2019	1	1	1	1	1	5
Olie *et al.* [29]	2024	1	1	1	1	1	5
Chaudhary *et al.* [30]	2018	1	1	1	1	1	5
Dutta *et al.* [31]	2022	1	1	1	1	1	5
Rajial *et al.* [32]	2022	1	1	1	1	1	5
Sutinen *et al.* [33]	1998	1	1	1	1	1	5
Pemde *et al.* [34]	1996	1	1	1	1	1	5
Dubos *et al.* [35]	2008	1	1	1	1	1	5
El Shorbagy *et al.* [36]	2018	1	1	1	1	1	5
Ibrahim *et al.* [37]	2011	1	1	1	1	1	5
Babenko *et al.* [38]	2021	1	1	1	1	1	5
Gowin *et al.* [39]	2016	1	1	1	1	1	5
Gao *et al.* [40]	2024	1	1	1	1	1	5
Manyelo *et al.* [41]	2019	1	1	1	1	1	5
Sanaei Dashti *et al.* [42]	2017	1	1	1	1	1	5
Saleh *et al.* [43]	2020	1	1	1	0	1	4
Chen *et al.* [44]	2009	1	1	1	1	1	5
Debray *et al.* [45]	2019	1	1	1	1	1	5
Hou *et al.* [46]	2023	1	1	1	1	1	5
Mohamed *et al.* [47]	2012	1	1	1	1	1	5
Fu *et al.* [49]	2014	1	1	1	1	1	5
Wang *et al.* [50]	2011	1	1	1	1	1	5
Carrol *et al.* [51]	2009	1	1	1	1	1	5
Irwin *et al.* [52]	2012	1	1	1	1	1	5
Ubenauf *et al.* [53]	2007	1	1	1	1	1	5
Kandil *et al.* [54]	2018	1	1	1	1	1	5
Knudsen *et al.* [55]	2007	1	1	1	1	1	5
Kalchev *et al.* [56]	2021	1	1	1	1	1	5
Mentis *et al.* [57]	2016	1	1	1	1	1	5
Peng *et al.* [58]	2014	1	1	1	1	1	5
Zhang *et al.* [59]	2017	1	1	1	1	1	5

Ref, reference, C1, criterion 1 – outcome measures; C2, criterion 2 – background/literaturę review; C3, criterion 3 – sample; C4, criterion 4 – study design or methodology; C5, criterion 5 – conclusions; results of 4 or 5 mean high quality, result of 3 - moderate quality, and results of 0, 1 or 2 - low quality.
